# Don’t miss the diagnosis of sepsis!

**DOI:** 10.1186/s13054-014-0529-6

**Published:** 2014-09-27

**Authors:** Paul E Marik

**Affiliations:** Division of Pulmonary and Critical Care Medicine, Eastern Virginia Medical School, Norfolk, VA 23507 USA

## Abstract

The early detection and treatment of sepsis are the most important factors in improving the outcome of patients with this condition. However, many patients admitted to hospital experience a long delay in the diagnosis of sepsis. Furthermore, it is not uncommon for febrile patients to be sent home from the Emergency Department or the physician’s office with the diagnosis of ‘flu’ only to return hours or days later in overt septic shock. The early diagnosis of sepsis may be challenging as many of the signs and symptoms are non-specific. Clinical studies suggest that early diagnosis of sepsis requires a high index of suspicion and comprehensive clinical evaluation together with laboratory tests, including a complete blood count with differential, lactate and procalcitonin levels.

## Introduction

Sepsis is amongst the most common reasons for admission to ICUs throughout the world. The early detection and timely administration of appropriate antibiotics are the most important factors in improving the outcome of patients with sepsis. However, the initial signs and symptoms of sepsis are frequently non-specific, leading to a delay in diagnosis. Furthermore, the diagnostic characteristics of the systemic inflammatory response syndrome (SIRS) are not useful in distinguishing infectious from non-infectious causes of SIRS. An elevated white blood count, neutrophilia or eosinopenia are frequently used to diagnose bacterial sepsis; however, these variables have low diagnostic value.

## Biomarkers to diagnose sepsis

Blood cultures are considered the clinical gold standard for the diagnosis of bacterial infections. However, blood cultures are only positive in 20 to 30% of patients with sepsis; moreover, it takes 2 to 3 days before the results become available. As the clinical diagnosis of sepsis can be challenging and microbiological tests are unhelpful, several biomarkers have been developed to assist in the early diagnosis of sepsis, including procalcitonin (PCT), C-reactive protein (CRP) and, more recently, circulating cell-free DNA (cfDNA). In a well conducted study reported in a previous edition of *Critical Care*, Garnacho-Montero and colleagues [1] investigated the role of these biomarkers in distinguishing infectious from non-infectious SIRS. They demonstrated that PCT had excellent diagnostic accuracy (area under curve (AUC) 0.87; 95% confidence interval (CI) 0.81 to 0.94), that for CRP was modest (AUC 0.69; 95% CI 0.59 to 0.79) while that for cfDNA was poor (AUC 0.5; 95% CI 0.61 to 0.71). The findings of this study are remarkably similar to the results of a recent meta-analysis performed by Wacker and colleagues [2]. In this meta-analysis the sensitivity of PCT for the diagnosis of sepsis was 0.77 (95% CI 0.72 to 0.81), the specificity was 0.79 (95% CI 0.74 to 0.84) and the area under the receiver operator characteristic curve was 0.85 (95% CI 0.81 to 0.88). Tromp and colleagues [3] studied a panel of biomarkers in patients presenting to the Emergency Department with suspected sepsis. In this study PCT had the best predictive value for bacteremia (AUC 0.80). Similarly, Su and colleagues [4] evaluated 32 clinical signs, symptoms and laboratory tests available during a patient’s stay in the Emergency Department that were predictive of bacteremia. In this study, PCT was the variable with the best diagnostic accuracy. Furthermore, thrombocytopenia, lymphocytopenia and bandemia were also predictive of bacteremia. Additional studies have confirmed that bandemia has a high predictive value for the diagnosis of sepsis [5]. Bacterial sepsis is typically characterized by neutrophilia and lymphocytopenia. While the total white blood cell count and neutrophil count are poor predictors of sepsis [4,5], an increased neutrophil to lymphocyte count ratio has been shown to be a useful marker of sepsis [6]. Molecular methods based on polymerase chain reaction technology are currently being investigated and hold promise for the early diagnosis of bacterial infection and pathogen identification [7,8].

The use of PCT for the diagnosis of sepsis is controversial [9]; however, clinical studies suggest that PCT is currently the most useful biomarker to aid in its diagnosis. In healthy individuals, PCT levels are very low (<0.01 ng/ml) while in patients with bacterial sepsis the levels increase dramatically, sometimes to more than several hundred nanograms per milliliter. A PCT level >0.5 ng/ml is highly suggestive of a bacterial infection while a level <0.1 ng/ml makes this diagnosis less likely [10]. However, the optimal diagnostic threshold is unclear and has been reported to vary from 0.25 to 1.4 ng/ml [3,10]. This variation in diagnostic threshold may partly be explained by the case mix of each study and the fact that patients with Gram-negative infection have significantly higher PCT levels than those with Gram-positive infections [11-13]. Infection with a Gram-negative pathogen is highly likely in a patient with a PCT level >5 ng/ml. It should be noted that patients with fungal infections usually have much lower or ‘normal’ PCT level [11].

It is important to emphasize that the PCT assay can yield both false positive and false negative results. Furthermore, there is no perfect ‘sepsis test’. The diagnosis of sepsis requires a high index of suspicion. However, one or more of the parameters listed in Table 1 should increase the diagnostic likelihood of sepsis. These parameters are readily available on admission to the hospital or in the Emergency Department and should be obtained to support the diagnosis of sepsis. In many patients who present to the Emergency Department the diagnosis of sepsis is obvious - high fever, high white blood count and an obvious source of infection. However, not uncommonly patients with sepsis may present with vague constitutional symptoms, mild hypotension and tachycardia or with a fever and myalgia that are attributed to ‘a viral syndrome’. These patients should not be sent home without further workup, unless they obviously have a viral syndrome and epidemiological data support the diagnosis of influenza.

**Table 1:** **Diagnostic features suggestive of sepsis**

**Diagnostic criteria**	**Threshold**
**Fever**	>38.3°C
**Tachycardia**	>120/minute
**Systolic blood pressure**	<90 mmHg
**Procalcitonin**	>0.5 ng/ml
**Bandemia**	>5%
**Lymphocytopenia**	<0.5 × 10^3^ ul
**or neurophil/lymphocyte ratio**	>10
**Thrombocytopenia**	<150 × 10^3^ ul
**Lactate**	>2.0 meq/l

## Conclusion

When the diagnosis of sepsis is not clear we recommend a complete blood count with differential, blood lactate level and PCT as well as appropriate bacteriological cultures.

## Abbreviations

AUC: Area under curve
cfDNA: Circulating cell-free DNA
CI: Confidence interval
CRP: C-reactive protein
PCT: Procalcitonin
SIRS: Systemic inflammatory response syndrome
